# Impact of Pulse Wave Velocity and Parameters Reflecting Android Type Fat Distribution on Left Ventricular Diastolic Dysfunction in Patients with Chronic Coronary Syndromes

**DOI:** 10.3390/jcm9123924

**Published:** 2020-12-03

**Authors:** Marlena Paniczko, Małgorzata Chlabicz, Jacek Jamiołkowski, Paweł Sowa, Małgorzata Szpakowicz, Magda Łapińska, Marcin Kondraciuk, Katarzyna Ptaszyńska-Kopczyńska, Andrzej Raczkowski, Anna Szpakowicz, Karol Adam Kamiński

**Affiliations:** 1 Department of Population Medicine and Civilization Diseases Prevention, Medical University of Bialystok, 15-269 Bialystok, Poland; m.paniczko@gmail.com (M.P.); mchlabicz@op.pl (M.C.); jacek909@wp.pl (J.J.); sowa@umb.edu.pl (P.S.); malgorzata.szpakowicz@umb.edu.pl (M.S.); magda.lapinska@umb.edu.pl (M.Ł.); marcin.kondraciuk@umb.edu.pl (M.K.); andrzej.raczkowski@umb.edu.pl (A.R.); 2 Department of Invasive Cardiology, Medical University of Bialystok, 15-269 Bialystok, Poland; 3 Department of Cardiology, Medical University of Bialystok, 15-269 Bialystok, Poland; kasia.ptaszynska@op.pl (K.P.-K.); akodzi@poczta.onet.pl (A.S.)

**Keywords:** left ventricular diastolic dysfunction, android fat distribution, chronic coronary syndromes

## Abstract

Background: Left ventricular diastolic dysfunction (LVDD) is caused by a decreased left ventricle relaxation and is associated with an increased risk of symptomatic heart failure (HF) and excessive mortality. Aim: To evaluate the frequency and factors related to LVDD in the population with chronic coronary syndromes (CCS). Methods: 200 patients (mean age 63.18 ± 8.12 years, 75.5% male) with CCS were included. LVDD was diagnosed based on the recent echocardiography guidelines. Results: LVDD was diagnosed in 38.5% of CCS population. From the studied factors, after adjustment for age, sex, and N-terminal pro-brain natriuretic peptide (NT-proBNP), LVDD associated positively with android/gynoid (A/G) fat mass ratio, left ventricular mass index (LVMI), and negatively with Z-score and left ventricular ejection fraction (LVEF). In stepwise backward logistic regression analysis, the strongest factors associated with LVDD were pulse wave velocity value, handgrip strength and waist to hip ratio (WHR). Conclusions: LVDD is common among CCS patients and it is associated with parameters reflecting android type fat distribution regardless of NT-proBNP and high-sensitivity troponin T concentrations. Deterioration in diastolic dysfunction is linked with increased aortic stiffness independently of age and sex. Further studies evaluating the effects of increasing physical fitness and lowering abdominal fat accumulations on LVDD in CCS patients should be considered.

## 1. Introduction

Left ventricular diastolic dysfunction (LVDD) is caused by a decreased left ventricle (LV) relaxation or increased LV stiffness [1]. In early LVDD, elevated LV stiffness is associated with diastolic filling abnormalities and normal exercise tolerance. Asymptomatic LVDD may be present for long periods before it develops into a symptomatic phase. When the disease progresses, pulmonary pressures increase abnormally during exercise, causing reduced exercise tolerance. When filling pressures increase further, clinical signs of heart failure (HF) appear [2]. Asymptomatic mild LVDD is found in 21% of the population, moderate or severe LVDD is present in 7% and is associated with an increased risk of symptomatic HF and mortality [3]. This asymptomatic period represents a potential time to intervene to prevent symptomatic HF. Major risk factors for diastolic dysfunction in the general population include age, obesity, diabetes mellitus, hypertension, and LV hypertrophy [4,5]. Chronic coronary syndromes (CCS) are one of the forms of the coronary artery disease (CAD), characterized by accumulation of the atherosclerotic plaques in epicardial coronary arteries. They exclude situations in which an acute coronary artery thrombosis dominates the clinical presentation (which are acute coronary syndromes) [6]. CAD is commonly listed as a mechanism underlying LVDD, as myocardial ischemia may induce impaired relaxation [7], but according to recent data, stable CAD was not independently associated with LVDD [8], probably because many of the same factors contribute to atherosclerosis may also result in LVDD e.g., hypertension and vascular stiffening.

The prevalence of LVDD among individuals with well-documented CCS is not exactly known, nor the factors that contribute to this phenomenon. We aimed to evaluate the frequency and factors related to left ventricular diastolic dysfunction (LVDD) in the population with chronic coronary syndromes (CCS).

## 2. Methods

### 2.1. Study Population

The study was conducted in 2016–2018 on patients with CCS aged between 41 and 79. The study sample consisted of 257 patients hospitalized in three local hospitals for coronary events that occurred 12–18 months before the date of the study entry. The reasons for hospitalization were elective percutaneous coronary intervention (PCI) 96 (37.4%), acute myocardial infarction with ST-segment elevation (STEMI) 65 (25.3%), 71 (27.6%) acute myocardial infarction with non-ST-segment elevation (NSTEMI), and 25 (9.7%) unstable angina/acute myocardial ischemia. Due to the variability of the cycle length, the absence of organized atrial function and the frequent occurrence of the LA enlargement regardless of the filling pressure, the assessment of diastolic function by the Doppler method in patients with atrial fibrillation (AF) is severely limited [9]. For this reason or the lack of complete echocardiography (ECHO) data, 57 patients were excluded from the research group. As a result, 200 individuals (151 men and 49 women) were included in the study.

### 2.2. Data Collection and Assays

The details of the participants’ medical history were collected from questionnaires at the time of the study entry. All study patients underwent a laboratory assessment and physical examination. Peripheral intravenous fasting blood samples were collected at the time of a visit, which always took place in the morning. The anthropometric measurements including height, weight, circumferences of waist, abdomen, and hips were taken. Body mass index (BMI) was calculated as weight in kilograms divided by height in meters squared. Waist to hip ratio (WHR) was calculated as a ratio between waist and hips circumference. According to WHO guidelines, abdominal obesity was defined as WHR ≥ 0.85 for women, ≥ for 0.9 men [10]. Blood pressure (BP) was measured using the oscillometric method (Omron Healthcare Co. Ltd. MG Comfort device) after the participants were seated for at least 5 min. Resting ECG was performed using the AMEDTEC ECGpro CardioPart 12 USB (AMEDTEC Medizintechnik Aue GmbH, Aue, Germany).

In ECHO, the measurements of the dimensions of interventricular septal thickness (IVST), left ventricular internal dimension (LVID), left ventricle posterior wall thickness (LPWT), left atrial (LA) volume and left ventricular ejection fraction (LVEF) using the Biplane method were performed [11]. The left ventricular mass (LVM) was calculated using the Devereux Formula [12] LVM  =  0.8(1.04(IVST  +  LVID  +  LPWT)^3^ − (LVID)^3^  +  0.6). Body surface area (BSA) was calculated by the formula: BSA = (W − 60) × 0.01 + H, where BSA is the body surface area in m^2^, W is the weight in kilograms and H is height in meters [13]. The left ventricular mass index (LVMI) was calculated by the formula LVM/BSA (LVMI_BSA_) [12] likewise by the formula LVM/height in m^2.7^ [11]. The left ventricular hypertrophy (LVH) was defined as LVMI_BSA_ ≥115 g/m^2^ for men and ≥95 g/m^2^ for women (LVH_BSA)_ or LVH_height_ defined as LVMI_height_ ≥ 50 g/m^2.7^ for men and ≥ 47 g/m^2.7^ for women. The left atrial volume index was calculated by the formula LA volume/BSA (LAVI). The transmitral early diastolic velocity (E), peak velocity flow in late diastole caused by atrial contraction (A), and their deceleration time were acquired in the apical four-chamber view using pulse-wave Doppler at the level of the mitral valve tips during diastole. The early diastolic mitral annular tissue velocity (e’) was calculated as the average of septal and lateral mitral annular velocities, and E/e’ was calculate. Based on the EACVI/ASE 2016 recommendations, transmitral E and A waves velocities, septal, and lateral E’ tissue velocities, indexed left atrium volume and peak velocity of tricuspid regurgitation, depending on the LVEF were used to define LVDD [9]. The algorithm for the evaluation and qualification of participants to the group with the LVDD is presented in Table 1. For the purpose of this study LVDD was redefined by fusing the categories “indeterminate” and “abnormal”.

Artery stiffness assessment parameters, i.e., carotid-femoral pulse wave velocity (PWV), augmentation index (AIx), and central pressure (CP) were measured by the applanation tonometer and an oscillometric measurement (SphygmoCor XCEL) in a supine position preceded by a 10-min rest. Increased arterial stiffness was defined by PWV ≥ 10 m/s, independently of age [14].

Body composition was measured by the dual energy X-ray absorptiometry (DEXA) (GE Healthcare, Chicago, IL, USA) with total body mass divided into 3 compartments: fat, lean and bone mass. Fat, lean, and bone mass index was calculated as fat, lean and bone mass in kilograms divided by height in meters squared. The gynoid (G) and android (A) fat were measured automatically. The android region is the area between the ribs and the pelvis, totally enclosed by the trunk region. The upper demarcation is 20% of the distance between the iliac crest and the neck. The lower demarcation is at the top of the pelvis. The gynoid region includes the hips and upper thighs and overlaps both the leg and trunk regions. The upper demarcation is below the top of the iliac crest at a distance of 1.5 times the android height. The total height of the gynoid region is 2 times the height of the android region [15]. The A/G ratio was calculated between the fat of the android (central) and fat of the gynoid (hip and thigh) regions. The bone density index referring to a representative young population expresses a T-score, while referring to a control group of the same age, sex, and race, and additionally corrected for body weight Z-score values [16]. Grip strength was expressed in kg and measured using a digital hand dynamometer (SAEHAN DHD-1, Saehan Corporation, Masanhoewon-Gu Gyeongsangnamdo, Korea). Measurements were obtained in standardized conditions, with the participants in a seated position, an elbow at 90° and a handle adjusted to the second position. Having received explanation on the procedures, and after familiarization with the instrument, the patients were asked to apply the maximum hand grip strength for 3 to 5 s. The procedure was performed three times with each hand alternately, with an interval of one minute between each measurement. The maximum value was used for the analysis. Handgrip strength as a dichotomous variable was obtained referring to the reference values presented by Wang, Y.C. [17] taking into account gender, age groups, and the maximum handgrip value of the dominant hand for the 10th, 25th, and 50th percentiles (analyses in Supplementary Materials).

The concentrations of N-terminal pro-brain natriuretic peptide (NT-proBNP) and high-sensitive troponin T (hs-TnT) were determined by the electrochemiluminescence method on the Cobas e411 from ROCHE. The analytical measurement range for NT-proBNP was 5–35.000 pg/mL, and 3–10.000 pg/mL for hs-TnT. The concentrations of NT-proBNP and hs-TnT below the detection threshold were taken as 50% of the minimum measurement range. Glucose concentration was determined by enzymatic method with hexokinase on a Cobas C111 analyzer from ROCHE. For the determination of insulin, we used manual kits DiaSource by immunoradiometric method (IRMA).

Diabetes mellitus was defined as serum fasting glucose level ≥126 mg/dL or 2-h glucose ≥200 mg/dL, or the history of diabetes mellitus or use of hypoglycemic agents.

### 2.3. Ethical Issues

Ethical approval for this study was provided by the Ethics Committee of the Medical University of Bialystok (Poland) on 29 September 2016 (approval number: R-I-002/323/2016). The study was conducted in accordance with the Declaration of Helsinki and all participants gave a written informed consent.

### 2.4. Statistical Analysis

Descriptive statistics for quantitative variables were presented as means and standard deviations and as counts and frequencies for qualitative variables. Comparisons of continuous variables between subgroups were conducted using Mann–Whitney or Fisher’s tests. Associations between LVDD and other clinical and biochemical variables were analysed using simple and multiple logistic regression models. Multiple regression models where adjusted for age, sex (Model 1), for age, sex, NT-proBNP (Model 2), and for age, sex, NT-proBNP, hs-TnT (Model 3), for LVMI_BSA_ (Model 4) and for LVH_BSA_ (Model 5). Logistic regression models were presented using odds ratio and confidence intervals. Simple and multiple linear regression models were employed to identify the determinants of PWV value. Statistical hypotheses were verified at 0.05 significance level. The Statistica 13.1 software (StatSoft Polska, Cracow, Poland) was used for all calculations.

## 3. Results

The baseline characteristics of the study population are shown in Table 2. The mean age was 63.18 ± 8.12 years and 75.5% of participants were male. Overall, 39% of patients were obese (BMI ≥ 30 kg/m^2^) and 42.5% were overweight (BMI ≥ 25 kg/m^2^ and BMI < 30 kg/m^2^), and 77 participants (38.5%) were diagnosed with LVDD using ECHO.

The comparison of groups with and without LVDD is presented in Table 3. There were no differences in age (*p* = 0.121), gender (*p* = 1.000), or BMI (*p* = 0.391). The individuals with LVDD had higher WHR index (*p* = 0.046) and were more often diabetic (*p* = 0.026). The PWV value was significantly higher (*p* = 0.003) in the group with LVDD, but there were no differences between the groups in terms of blood pressure (BP), central blood pressure (CBP), and augmentation index. In the body composition analysis, only Z- score value was lower in the group with LVDD. In laboratory test, higher level of NT-proBNP (*p* < 0.001), hs-TnT (*p* = 0.018) and 120min glucose (*p* = 0.043) in oral glucose tolerance test (OGTT) were related with LVDD. In ECHO, LVEF was lower in the LVDD group (*p* < 0.001), while LVMI_BSA_, LVMI_height_ and LAVI had higher values in the group with LVDD (*p* < 0.001, *p* = 0.001, *p* = 0.034, respectively).

The PWV determinants in the study population analysis was presented in Supplementary Materials.

On the multivariable logistic regression analysis, the variables that remained associated with LVDD in Model 1, after adjusting for age and gender, were levels of NT-proBNP and hs-TnT, WHR, Z-score, PWV value, LVMI_BSA_, LVMI_height_, and LVEF (Table 4). In addition, a new parameter appeared, a lower value of handgrip strength which was significantly correlated with LVDD.

The relationship between LVDD and the studied factors changed after adjustment for age, sex and NT-proBNP in Model 2. LVDD was correlated with lower value of Z-score bone density, LVMI_BSA_, LVMI_height_, LVEF, and android type obesity (higher A/G fat mass ratio). The higher A/G fat mass ratio remained positively associated with LVDD even after adjusting for hs-TnT in Model 3 (Table 5).

An analysis was also performed between the LVDD and the examined factors, in which the model was corrected for LVMI (Model 4) and LVH (Model 5). The results were presented in Supplementary Materials.

In logistic regression analysis with stepwise elimination of variables, the strongest factors associated with LVDD were WHR, PWV value and handgrip strength (Table 6).

## 4. Discussion

The present study reports on the frequency of LVDD in population with CCS and provides evidence that the parameters reflecting android type fat distribution is associated with LVDD after correction for biochemical markers of cardiac dysfunction. Lower muscle strength and lower bone density are also related to this phenomenon suggesting the important role of physical fitness in both these variables. Moreover, PWV value—a variable presenting stiffness of large arteries—positively associated with the presence of LVDD. We showed that the effect of abdominal adiposity on LVDD was more powerful than general adiposity.

Venkataraman [8] showed that 9.6% of participants with low-intermediate risk of coronary artery disease (CAD) had LVDD, and atherosclerosis was not directly associated with subclinical LV dysfunction. In present study, all participants had CAD, the population was older, BMI was higher and LVEF was lower compared to the above mentioned study. In the study of Japanese patients with suspected CAD, mean age was 66 ± 13 years. LVDD was assessed based on the 2016 EACVI/ASE algorithm and was recognised in 71 participants (28.2%) [18]. Consequently, this frequency was lower than in the presented study (38.5%). According to Kuznetsova [19], the incidence of LVDD in the general population was 27.3%; it increased with age, body mass index, serum insulin, serum creatinine, and NT-proBNP. However, in the aforementioned study, the assessment of LVDD was based only on the transmitral, pulmonary blood flows and the tissue doppler imaging of mitral annular velocities, not on the currently accepted criteria. Another study, comparing LVDD assessment guidelines form 2009 and 2016, showed that in the general population, with a mean age of 62 ± 10.5 years, the prevalence of LVDD and indeterminate diastolic dysfunction assessed according to the latest recommendations was 16.6% [20]. Furthermore, in the other study based on patients older than 50 years without documented cardiovascular disease, LVDD was defined as septal annular peak velocity (e’) < 8 cm/s and LAVI ≥ 34 mL/m2. The prevalence of LV diastolic dysfunction was high and amounted to 63.5% [21]. Moreover, researchers showed differences in age and BMI between groups with and without LVDD as opposed to our study. There were no age, gender, blood pressure, or BMI differences between population with and without LVDD, similarly to the population with low–intermediate risk of CAD [8]. Our findings which demonstrate a correlation between the presence of LVDD and LVMI are in line with the earlier studies [4,19,22].

Major risk factors for diastolic dysfunction include age, diabetes mellitus, hypertension, and LV hypertrophy [5]. LVDD is common in diabetic patients and may be diagnosed in every third case (34%). It is associated with increased LVM, wall thickness and arterial stiffness [5,23]. In the current study, we observed more frequent diabetes mellitus and higher concentration of glucose in the 120th minute of OGTT in patients with LVDD. However, in the multivariable analysis, after adjustment for NT-BNP or troponin concentrations, these parameters were no longer relevant. In another study of people with diabetes mellitus, without overt heart disease, the percentage of patients with diastolic dysfunction increased from 49% at the baseline to 67% during a three-year follow-up period. Older age and elevated blood pressure over time were associated with an increased risk of LVDD [24].

### 4.1. Impact of Fat Tissue Distribution on LVDD

Multiple studies have confirmed overweight/obesity as a risk factor for the development of HF [25]. In publications, the most commonly used indicator was BMI. Differently from the described observations, Russo et al. [26] have shown that increased BMI was significantly associated with increased risk of diastolic dysfunction independent of LV mass in a high-risk population without evidence of heart disease. Several studies reported a strict relationship between obesity and LVDD [4,27]. In the population of 769 elderly people participating in the Baltimore Longitudinal Study of Aging, the degree of obesity was defined by BMI and waist circumference to assess central adiposity. The author’s suggested that the effect of central adiposity on LV diastolic function was independent of general adiposity [22]. A growing body of research indicates that central obesity may play a pivotal role in obesity-related changes in cardiac function and structure. Among numerous variables describing obesity used in this study, the increased WHR and A/G ratio, describing central obesity, were the most influential on LVDD. A strong relationship between WHR and LVDD has been demonstrated previously in a study of people over 45 in Olmsted County, Minnesota, USA, in which WHR had a stronger association with LV diastolic function then waist circumference and BMI. For each standard deviation increase in WHR, the odds of LVDD increased 1.55 times [28]. Yaylalı [29], on the other hand, stated that WHR had no effect on LVDD after adjustment for age and gender. In our study, the effect of WHR was even more pronounced when adjusted for age and gender. Its effect disappeared after adjustment for NT-BNP or troponin concentrations, but simultaneously in those analyses, a different parameter reflecting abdominal type fat distribution emerged—a ratio of android and gynoid fat. Nevertheless, WHR was one of the two variables independently associated with the presence of LVDD in the logistic regression. The differences may be due to the small sample size and characteristics of the population (without any cardiovascular risk factors) in the Yaylalı YT study. A study of the European populations on various obesity indicators as predictors of cardiovascular mortality showed that WHR was a stronger predictor for CVD mortality than BMI [30]. This data may offer mechanistic insight into why central obesity and reduced physical fitness is associated with worse cardiac outcomes, including LVDD.

### 4.2. Impact of Reduced Physical Fitness on LVDD

We found evidence to support the value of maximum handgrip strength as a marker associated with cardiac dysfunction in the studied population. This value represents the maximum strength of hand and forearm and can be used as an indicator of general muscle strength, which is dependent on the general physical fitness. There are a number of studies indicating the relationship between handgrip strength and cardiovascular disease [31,32]. The potential pathophysiological mechanisms underlying grip strength and the incidence of CVD are not thoroughly understood. To the best of our knowledge, this study is the first to look at handgrip strength as a predictor for LVDD. The study on the relationship between handgrip and measures of cardiac structure and function show that higher levels of handgrip strength were associated with a pattern indicative of less cardiac hypertrophy and remodeling [32]. In our study, we have proved that lower handgrip strength, as a surrogate of reduced physical fitness, increases the risk of LVDD in the CCS population.

The positive effect of physical activity and fitness on bones has been shown in the study presenting the relationship between handgrip strength and bone mineral density [33]. Wang et al. [34] evaluated the association between BMD and LV diastolic function in men with type 2 diabetes mellitus. The study showed that increasing bone density is associated with LVDD in diabetic men. However, no such relationship has been demonstrated for people without carbohydrate disorders. In hypertensive patients, reduced bone mineral density was associated with LVDD, but not with LV hypertrophy [35]. Our study showed a statistically significant association between bone density and LVDD after adjustment for gender or age.

### 4.3. Impact of Arterial Stiffness on LVDD

Several clinic-based population studies have reported a relationship between arterial stiffness and LVDD, indicating that arterial stiffness may increase pulse pressure and LV afterload, thus potentially contributing to the development of LVDD [36]. As for the method type, Tanaka et al. [37] conducted a comparative carotid-femoral PWV and brachial ankle PWV analysis and demonstrated that both of these measures are similarly associated with coronary heart disease risk factors and predict clinical events in the same range. In a healthy Korean population, the scientists observed that brachial-ankle PWV was independently associated with LV filling pressure after controlling for age, sex, and body mass index [38]. The study implicating the usefulness of brachial-ankle PWV as an indicator of LV diastolic function was conducted in Korean adults older than 50 years without documented cardiovascular disease. The results of the mentioned study showed that reduced arterial stiffness was independently associated with normal diastolic function [21]. An age-similar population to our study, was tested among Canberra residents and proven age-related deterioration of left ventricular diastolic dysfunction is independently related to increasing aortic stiffness. Carotid-femoral PWV has also been shown to be useful in identifying preclinical LVDD that outperformed central and brachial pulse pressure [39]. Tsai et al. [40] examined a group of patients with suspected coronary artery disease, heart failure, hypertension, abnormal cardiac physical examination, survey for dyspnea and the pre-operative cardiac function survey. He has proven that patients with higher brachial-ankle PWV had higher prevalence of LVDD. Our findings support the concept that increased aortic stiffness is associated with LVDD also in patients with chronic coronary syndromes.

In our study there were no statistical differences in BPs, BPd, CPs, and CPd between analysed groups, and in further analysis these parameters remained irrelevant in reference to LVDD.

### 4.4. Impact of Ejection Fraction and Left Ventricular Mass Index on LVDD

Surveillance studies have documented a constant increase in incidence of HF with preserved ejection fraction (HFpEF) definded as EF of ≥50% in a patient with HF symptoms [23]. Studies have demonstrated that HFpEF is as prevalent as heart failure with reduced ejection fraction (HFrEF) [41]. It is important to note that many patients with systolic dysfunction have some degree of concomitant diastolic dysfunction, and only in some patients with preserved ejection fraction may have some degree of systolic dysfunction diagnosed using more sophisticated methods (e.g., global strain measurements), which suggests the unclear relationship between systolic dysfunction and LVDD [42]. In the current study we confirm strong relationship between decreased LVEF and LVDD.

Increased LVMI leads to an increase of the myocardial mass/volume ratio, and the degree of LVH is the main determinant of chamber stiffness. The main reason of myocardial diastolic tissue distensibility is the structure and concentration of the collagen. Tissue stiffness is increased in CCS by reparative interstitial fibrosis or scar following myocardial infarction. Moreover, an increase in regional asynchrony of LV contraction and relaxation is a result of regional ischemia as well as of LVH and tissue fibrosis. Known factors extrinsic to the LV causing LVDD include increased central blood volume, which will increase left ventricular pressure without altering the LV pressure–volume relation, and ventricular interaction mediated by pericardial restraint, which may cause a parallel upward shift of the diastolic LV pressure–volume relation [43]. In our study we confirm strong relationship between LVMI and LVDD.

### 4.5. Impact of Biochemical Markers on LVDD

The association of LVDD with NT-proBNP and hsTNT was demonstrated in the current study population and is in line with earlier studies [44]. Contrary to the presented study, Nah et al. [45], showed that LVDD was not associated with higher NT-proBNP levels. The lack of relationship can be explained by different characteristics of the groups studied: participants were younger and less overweight and obese, and with preserved LV ejection fraction (≥50%). Moreover, in a healthy population NT-BNP levels may be inversely associated with parameters promoting LVDD like central obesity and, in particular, android to gynoid fat ratio [46]. The relationship between concentrations of NT-proBNP and increased assessed LV filling pressures was noted in ambulatory, clinically stable hypertensive patients [45]. Quiroga et al. [47] used among others NT-proBNP, hs-TnT and LVDD as markers associated with death and cardiovascular events in hemodialysis patients. It has been proven that cardiac biomarkers ensure good information for identifying high-risk patients, and LVDD is a long-term, independent predictor of mortality and development of cardiovascular events.

### 4.6. Limitation

The limitation of this study was a relatively small number of patients. However, homogeneity of presented population adds value to this study. We determined LVDD as a combination of the “indefinite” and “incorrect” categories, limiting our results to LVDD from mild to severe. We did not assess the physical activity directly, so we can only rely on indirect surrogates such as handgrip strength and bone density which may also depend on other factors. It is worth noting that all echocardiographic parameters recommended in the latest guidelines were used to define LVDD.

## 5. Conclusions

LVDD is common in patients with chronic coronary syndromes and may be diagnosed in nearly 4 out of 10 such patients. It is associated with parameters reflecting android type fat distribution and surrogates of reduced physical activity: lower muscle strength and lower bone density. Weight reduction and physical activity are indicated in the population with CCS. Moreover, our results suggest that deterioration in diastolic dysfunction is associated with increasing aortic stiffness independent of age and sex. The overall utility of PWV to identify diastolic disorder was superior to peripheral or central pressure. Therefore, screening patients by means of carotid-femoral PWV may be helpful in identifying the patients of LVDD.

## Figures and Tables

**Table 1:** Algorithm evaluation of LVDD [9].

Normal EF (≥50%)	1. Average E/e’ > 14 2. Septal e’ velocity < 7 cm/s or lateral e’ velocity < 10 cm/s 3. TR velocity _max_ > 2.8 m/s 4. LAVI > 34 mL/m^2^	1st criteria fulfilled			Normal LVDD
		2nd criteria fulfilled			Indeterminate LVDD
		3rd or 4th criteria fulfilled			Abnormal LVDD
Depressed EF (<50%)	E/A ≤ 0.8 + E ≤ 50 cm/s				Grade I LVDD
	E/A ≤ 0.8 + E > 50 cm/s or 0.8 < E/A < 2	When possible assessment of 3 criteria: 1. Average E/e’ >14 2. TR velocity >2.8 m/s 3. LAVI > 34 mL/m^2^	2 of 3 or 3 of 3 Negative		
			When only 2 criteria are available	2 negative	
				1 positive and 1 negative	Indeterminate LVDD
				1 negative	Grade II LVDD
			2 of 3 or 3 of 3 Positive		
	E/A ≥ 2				Grade III LVDD

LVDD, left ventricular diastolic dysfunction; EF, ejection fraction; E, peak early diastolic velocity; e’, early diastolic mitral annular tissue velocity; TR, tricuspid regurgitation; LAVI, left atrial volume index; A, peak late diastolic velocity; cm, centimeter; s, second; m, meter; ml, milliliter.

**Table 2:** Baseline characteristics of study population.

Variables	Value (*n* = 200)
Age, years	63.18 ± 8.12
Gender, male	151 (75.5)
BMI, kg/m^2^	29.47 ± 5.08
BMI 25–29.99 kg/m^2^	85 (42.5)
BMI ≥ 30 kg/m^2^	78 (39)
WHR	0.95 ± 0.08
WHR ≥ 0.85 women, ≥ 0.9 men	167 (83.5)
LV ejection fraction, %	52.24 ± 7.87
LVMI _BSA_, g/m^2^	111.79 ±28.86
LVMI _height,_ g/m^2.7^	52.73 ± 14.52
LAVI, mL/m^2^	25.03 ± 7.41
Diastolic dysfunction of left ventricle, *n*	77 (38.5)
NT-proBNP, pg/mL	271.30 ± 406.65
hs-TnT, pg/mL	12.53 ± 11.78
Creatinine, µmol/L	86.57 ± 19.56
GFR, mL/min/1.73m^2^	82.12 ± 22.49
Total cholesterol, mg/dl	159.42 ± 45.38
Triglycerides, mg/dl	130.63 ± 135.49
High-density lipoprotein cholesterol, mg/dl	52.06 ± 16.47
Low-density lipoprotein cholesterol, mg/dl	90.88 ± 37.20
Low-density lipoprotein cholesterol ≥55 mg/dl, *n*	177 (88.5)
Diabetes mellitus, *n*	78 (39)
Smoking history, *n*	148 (74)
Current smoking, *n*	46 (23)
Use of ACE-I/ARB, *n*	172 (86)
Use of beta-blockers, *n*	176 (88)
Use of statin, *n*	182 (91)

The data is shown as *n* (%), mean ± SD. SD, standard deviation; BMI, body mass index; kg, kilogram; m^2^, square meter; WHR, waist to hip ratio; LV, left ventricle; LVMI, left ventricular mass index; BSA, body surface area; g, gram; LAVI, left atrial volume index; mL, millilitre; NT-proBNP, N-terminal pro-brain natriuretic peptide; hs-TnT, high-sensitivity troponin T; GFR, glomerular filtration rate Cockcroft–Gault Equation; ACE-I, angiotensin-converting enzyme inhibitors; ARB, angiotensin II receptor blockers.

**Table 3:** Characteristics of the study population according to the presence of left ventricular diastolic function.

Variables	Study Population (*n* = 200)		
	Subjects without LVDD (*n* = 123)	Subjects with LVDD (*n* = 77)	*p* Values
Age, years	62.50 ± 7.72	64.26 ± 8.69	0.121
Gender, male	93 (75.6)	58 (75.3)	1.000
Diabetes mellitus	40 (33.1)	38 (49.4)	0.026
Ever smoking	90 (73.8)	58 (76.3)	0.739
Current smoking	30 (24.6)	16 (21.1)	0.607
BMI, kg/m^2^	29.18 ± 4.77	29.92 ± 5.58	0.391
BMI 25–29.99 kg/m^2^	54 (43.9)	31 (40.3)	0.661
BMI ≥ 30 kg/m^2^	47 (38.2)	31 (40.3)	0.882
WHR	0.94 ± 0.08	0.96 ± 0.08	0.046
WHR ≥ 0.85 women, ≥ 0.9 men	102 (82.9)	65 (84.4)	0.847
BPs, mmHg	133.66 ± 16.84	132.57 ± 21.25	0.512
BPd, mmHg	84.11 ± 10.49	83.22 ± 11.04	0.533
HR, bpm	65.31 ± 9.87	66.55 ± 10.36	0.418
CPs, mmHg	125.81 ± 14.73	124.60 ± 17.77	0.257
CPd, mmHg	81.19 ± 9.11	79.79 ± 10.68	0.201
Augmentation Index	12.63 ± 7.99	11.71 ± 8.74	0.309
PWV, m/s	8.64 ± 1.69	9.47 ± 1.87	0.003
PWV ≥ 10 m/s	22 (19.1)	23 (33.8)	0.033
T-score	0.08 ± 1.04	−0.29 ± 1.38	0.063
Z-score	0.19 ± 1.04	−0.11 ± 0.994	0.050
Handgrip strength _max_, kg	41.11 ± 11.41	38.01 ± 11.09	0.066
Fasting glucose, mg/dL	112.19 ± 41.35	114.69 ± 28.60	0.059
120 min glucose, mg/dL	128.59 ± 41.47	144.95 ± 43.94	0.043
Fasting insulin, mg/dL	15.89 ± 12.08	18.23 ± 17.08	0.465
120 min insulin, mg/dL	90.72 ± 71.48	112.59 ± 92.45	0.151
HOMA-IR	4.66 ± 4.85	5.70 ± 7.81	0.297
NT-proBNP, pg/mL	172.66 ± 203.00	430.45 ± 574.09	<0.001
hs-TnT, pg/mL	10.41 ± 4.86	15.92 ± 17.59	0.018
Creatinine, µmol/L	87.28 ± 18.14	85.43 ± 21.71	0.365
GFR, mL/min/1.73m^2^	80.83 ± 18.63	84.189 ± 27.59	0.682
LV ejection fraction, %	56.792 ± 4.723	45.10 ± 6.44	<0.001
LVMI _BSA_, g/m^2^	104.42 ± 25.00	123.51 ± 31.01	<0.001
LVMI _height,_ g/m^2.7^	48.86 ± 12.57	58.55 ± 15.37	<0.001
LVH _BSA_	38 (33.6)	47 (66.2)	<0.001
LVH _height_	57 (50.4)	50 (70.4)	0.009
E, m/s	0.70 ± 0.14	0.69 ± 0.19	0.418
A, m/s	0.66 ± 0.19	0.70 ± 0.21	0.167
E/A	1.12 ± 0.36	1.08 ± 0.56	0.063
Deceleration time, ms	203.31 ± 45.61	208.24 ± 60.09	0.731
TR velocity _max_, m/s	2.05 ± 0.54	1.84 ± 0.80	0.258
Septal e’ velocity, cm/s	7.29 ± 1.53	6.50 ± 2.11	<0.001
Lateral e’ velocity, cm/s	10.32 ± 3.74	8.79 ± 2.66	0.001
E/e’	8.28 ± 1.90	9.34 ± 2.95	0.024
LAVI, mL/m^2^	23.57 ± 5.81	27.11 ± 8.94	0.034

The data is shown as *n* (%), mean ± SD. SD, standard deviation; BMI, body mass index; kg, kilogram; m^2^, square meter; WHR, waist to hip ratio; BPs, systolic blood pressure; mmHg, millimeters of mercury; BPd, diastolic blood pressure; HR, heart rate; bpm, beats per minute; CPs, systolic central pressure; CPd, diastolic central pressure; PWV, pulse wave velocity; HOMA-IR, homeostasis model assessment of insulin resistance; NT-proBNP, N-terminal pro-brain natriuretic peptide; hs-TnT, high-sensitivity cardiac troponin T; GFR, glomerular filtration rate Cockcroft–Gault Equation; LV, left ventricle; LVMI, left ventricular mass index; BSA, body surface area; LVH, left ventricular hypertrophy; g, gram; E, peak velocity flow in early diastole caused by atrial contraction; A, peak velocity flow in late diastole caused by atrial contraction; ms, millisecond; TR, tricuspid regurgitation; e’, early diastolic mitral annular tissue velocity; LAVI, left atrial volume index.

**Table 4:** Univariable and multivariable predictors of left ventricular diastolic dysfunction.

Variables	Unadjusted Model		Model 1	
	OR (95%CI)	*p* Values	OR (95%CI)	*p* Values
Age, year	1.028 (0.991; 1.065)	0.137	-	-
Gender, male	1.016 (0.524; 1.968)	0.964	-	-
NT-proBNP, pg/mL	1.003 (1.001; 1.004)	<0.001	1.003 (1.001; 1.004)	<0.001
hs-TnT, pg/mL	1.068 (1.017; 1.121)	0.008	1.064 (1.010; 1.122)	0.020
BMI, kg/m^2^	1.030 (0.974; 1.089)	0.301	1.030 (0.974; 1.090)	0.304
WHR *	1.395 (0.950; 2.049)	0.090	1.697 (1.039; 2.771)	0.035
BPs, mmHg	0.997 (0.981; 1.012)	0.690	0.995 (0.979; 1.011)	0.540
BPd, mmHg	0.992 (0.966; 1.019)	0.567	0.995 (0.967; 1.024)	0.733
A/G fat mass*	1.088 (0.956; 1.239)	0.203	1.136 (0.973; 1.326)	0.106
T-score	0.768 (0.599; 0.985)	0.038	0.773 (0.595; 1.004)	0.053
Z-score	0.754 (0.564; 1.008)	0.056	0.718 (0.534; 0.966)	0.029
Handgrip strength _max_, kg	0.976 (0.950; 1.002)	0.069	0.956 (0.917; 0.997)	0.035
CPs, mmHg	0.995 (0.977; 1.014)	0.605	0.994 (0.976; 1.012)	0.510
CPd, mmHg	0.985 (0.956; 1.015)	0.328	0.989 (0.958; 1.020)	0.473
Augmentation Index	0.986 (0.951; 1.022)	0.449	0.980 (0.944; 1.018)	0.300
PWV, m/s	1.311 (1.094; 1.572)	0.003	1.296 (1.069; 1.571)	0.008
Diabetes mellitus	1.973 (1.098; 3.544)	0.023	1.858(1.024; 3.368)	0.041
120 min glucose, mg/dL	1.009 (1.000; 1.018)	0.047	1.008 (0.999; 1.017)	0.071
HOMA-IR	1.028 (0.979; 1.079)	0.273	1.030 (0.980; 1.083)	0.243
LV ejection fraction, %	0.532 (0.436; 0.649)	<0.001	0.475 (0.371; 0.607)	<0.001
LVMI _BSA_, g/m^2^	1.026 (1.013; 1.038)	<0.001	1.026 (1.013; 1.039)	<0.001
LVMI _height,_ g/m^2.7^	1.055 (1.029; 1.081)	<0.001	1.053 (1.028; 1.081)	<0.001

OR, odds ratio; CI, confidence interval; * per 0.1 units; NT-proBNP, N-terminal pro-brain natriuretic peptide; hs-TnT, high-sensitivity cardiac troponin T; BMI, body mass index; kg, kilogram; m^2^, square meter; WHR, waist to hip ratio; BPs, systolic blood pressure; mmHg, millimeters of mercury; BPd, diastolic blood pressure; HR, heart rate; bpm, beats per minute; A/G, android fat mass/gynoid fat mass; CPs, systolic central pressure; CPd, diastolic central pressure; PWV, pulse wave velocity; g, gram; HOMA-IR, homeostasis model assessment of insulin resistance; LVMI, left ventricular mass index; BSA, body surface area; Model 1: adjusted for age and sex.

**Table 5:** Multivariable predictors of left ventricular diastolic dysfunction.

Variables	Model 2		Model 3	
	OR (95%CI)	*p* Values	OR (95%CI)	*p* Values
BMI, kg/m^2^	1.052 (0.988; 1.119)	0.112	1.049 (0.984; 1.117)	0.140
WHR *	1.675 (0.994; 2.825)	0.053	1.551 (0.906; 2.655)	0.109
BPs, mmHg	0.997 (0.980; 1.014)	0.696	0.996 (0.980; 1.014)	0.684
BPd, mmHg	0.999 (0.970; 1.029)	0.957	0.996 (0.967; 1.026)	0.799
A/G fat mass *	1.219 (1.031; 1.441)	0.021	1.211 (1.019; 1.438)	0.030
T-score	0.820 (0.620; 1.084)	0.163	0.828 (0.624; 1.100)	0.193
Z-score	0.727 (0.530; 0.998)	0.048	0.746 (0.542; 1.027)	0.072
Handgrip strength _max_, kg	0.964 (0.923; 1.007)	0.099	0.967 (0.926; 1.010)	0.135
CPs, mmHg	0.944 (0.975; 1.013)	0.533	0.993 (0.973; 1.013)	0.993
CPd, mmHg	0.992 (0.960; 1.025)	0.629	0.989 (0.957; 1.022)	0.514
Augmentation Index	0.972 (0.935; 1.011)	0.158	0.976 (0.938; 1.016)	0.239
PWV, m/s	1.201 (0.979; 1.473)	0.079	1.205 (0.978; 1.483)	0.079
Diabetes mellitus	1.665 (0.876; 3.165)	0.120	1.648 (0.857; 3.171)	0.134
120 min glucose, mg/dL	1.008 (0.999; 1.018)	0.085	1.009 (0.999; 1.019)	0.066
HOMA IR	1.048 (0.991; 1.109)	0.100	1.046 (0.988; 1.107)	0.121
LV ejection fraction, %	0.476 (0.370; 0.612)	<0.001	0.481 (0.374; 0.619)	<0.001
LVMI _BSA_, g/m^2^	1.018 (1.004; 1.032)	0.009	1.017 (1.004; 1.032)	0.014
LVMI _height,_ g/m^2.7^	1.040 (1.013; 1.067)	0.004	1.038 (1.011; 1.067)	0.006

OR, odds ratio; CI, confidence interval; * per 0.1 units; BMI, body mass index; kg, kilogram; m^2^, square meter; WHR, waist to hip ratio; BPs, systolic blood pressure; mmHg, millimeters of mercury; BPd, diastolic blood pressure; HR, heart rate; bpm, beats per minute; A/G, android fat mass/gynoid fat mass; CPs, systolic central pressure; CPd, diastolic central pressure; PWV, pulse wave velocity; g, gram HOMA-IR, homeostasis model assessment of insulin resistance; LVMI, left ventricular mass index; BSA, body surface area; Model 1: adjusted for age and sex; Model 2: model 1 + additional adjustment: NT-proBNP; Model 3: model 1 + additional adjustment: NT-proBNP and hsTNT.

**Table 6:** Results of stepwise backward logistic regression analysis of left ventricular diastolic dysfunction.

Variables	Full Model		Final Model	
	OR (95%CI)	*p* Values	OR (95%CI)	*p* Values
PWV, m/s	1.288 (1.048; 1.582)	0.016	1.262 (1.039; 1.533)	0.019
Handgrip strength _max_, kg	0.956 (0.912; 1.001)	0.057	0.962 (0.930; 0.995)	0.024
WHR *	1.591 (0.903; 1.135)	0.118	1.696 (1.029; 2.794)	0.038
Z-score	0.761 (0.543; 1.065)	0.111	-	-
Age, year	0.988 (0.940; 1.039)	0.639	-	-
Gender, male	1.092 (0.578; 2.063)	0.785	-	-

OR, odds ratio; CI, confidence interval; * per 0.1 units; PWV, pulse wave velocity; WHR, waist to hip ratio; kg, kilogram R^2^_Nagelkerke_ = 0.130; R^2^_Cox-Snell_ = 0.095.

## Supplementary Materials

The following are available online at https://www.mdpi.com/2077-0383/9/12/3924/s1, Table S1: Univariable and multivariable dichotomic predictors of left ventricular diastolic dysfunction, Table S2. Results of linear regression analysis of pulse wave velocity, Table S3. Multivariable predictors of left ventricular diastolic dysfunction.
