# Supplementary material for: Impact of Pulse Wave Velocity and Parameters Reflecting Android Type Fat Distribution on Left Ventricular Diastolic Dysfunction in Patients with Chronic Coronary Syndromes
Source: J Clin Med. 2020 Dec 3;9(12):3924. doi: 10.3390/jcm9123924 (PMC7761650; doi:10.3390/jcm9123924)
Supplement: Supplementary file 1 [file jcm-09-03924-s001.pdf]

Table S1. Univariable and multivariable dichotomic predictors of left ventricular diastolic dysfunction.

Variables	Unadjusted model		Model 1		Model 2		Model 3	
	OR (95%CI)	*p* values	OR (95%CI)	*p* values	OR (95%CI)	*p* values	OR (95%CI)	*p* values
$WHR_{\geq 0.85 \text{ women}, \geq 0.9 \text{ men}}$	1.115 (0.514; 2.419)	0.783	1.088 (0.469; 2.524)	0.845	1.075 (0.433; 2.672)	0.876	1.864 (0.340; 2.193)	0.758
PWV, ≥10 m/s	2.161 (1.090; 4.283)	0.027	1.980 (0.967; 4.054)	0.062	1.606 (0.735; 3.510)	0.235	1.625 (0.735; 3.593)	0.230
LVH_{BSA}	3.865 (2.063; 7.241)	<0.001	3.831 (2.023; 7.255)	<0.001	2.818 (1.434; 5.535)	0.003	2.891 (1.456; 5.739)	0.002
LVH_{height}	2.339 (1.247; 4.388)	0.008	2.236 (1.175; 4.257)	0.014	1.702 (0.862; 3.357)	0.125	1.701 (0.849; 3.408)	0.134
Handgrip strength $_{max, 10th}$	1.855 (0.189; 18.191)	0.596	1.801 (0.182; 17.783)	0.615	3.213 (0.248; 41.693)	0.372	2.947 (0.225; 38.569)	0.410
Handgrip strength $_{max, 25th}$	0.573 (0.225; 1.456)	0.242	0.610 (0.234; 1.595)	0.314	0.569 (0.204; 1.584)	0.281	0.525 (0.187; 1.476)	0.222
Handgrip strength $_{max, 50th}$	0.536 (0.287; 1.002)	0.051	0.576 (0.302; 1.098)	0.094	0.616 (0.307; 1.233)	0.171	0.611 (0.302; 1.234)	0.169

OR, odds ratio; CI, confidence interval; WHR, waist to hip ratio; PWV, pulse wave velocity; LVH, left ventricular hypertrophy; BSA, body surface area; Model 1: adjusted for age and sex; Model 2: model 1 + additional adjustment: NT-proBNP; Model 3: model 1 + additional adjustment: NT-proBNP and hsTNT;.

Table S2. Results of linear regression analysis of pulse wave velocity.

Variables	Unadjusted model			Model 1		
	B	R^2	*p* values	B	Adjusted R^2	*p* values
Age, year	0.075	0.109	<0.001	0.069	0.134	<0.001
Gender, male	−0.190	0.002	0.539	−0.032	0.043	0.921
NT-proBNP, pg/mL	0.001	0.068	<0.001	0.001	0.076	0.024
hs-TnT, pg/mL	0.024	0.026	0.030	0.020	0.056	0.109
BMI, kg/m^2	0.032	0.008	0.240	0.035	0.052	0.217
WHR	4.197	0.032	0.015	3.675	0.066	0.045
WHR, $_{\geq 0.85 \text{ women}, \geq 0.9 \text{ men}}$	0.392	0.007	0.264	0.321	0.048	0.368
BPs, mmHg	0.036	0.137	<0.001	0.035	0.173	<0.001
BPd, mmHg	0.022	0.017	0.085	0.022	0.060	0.087
A/G fat mass *	0.894	0.013	0.132	0.668	0.053	0.274

T- score	−0.040	0.001	0.721	−0.027	0.046	0.818
Z- score	−0.010	0.000	0.939	0.008	0.046	0.956
Handgrip strength $_{max}$, kg	−0.025	0.024	0.044	−0.025	0.056	0.050
CPs, mmHg	0.039	0.124	<0.001	0.037	0.154	<0.001
CPd, mmHg	0.033	0.033	0.014	0.029	0.069	0.034
Augmentation Index	0.040	0.035	0.011	0.048	0.093	0.003
Diabetes mellitus	0.695	0.036	0.011	0.621	0.069	0.030
120 min glucose, mg/dL	0.008	0.052	0.013	0.009	0.052	0.014
HOMA-IR	−0.010	0.001	0.645	−0.006	0.042	0.886
LV ejection fraction. %	−0.038	0.029	0.023	−0.022	0.033	0.252
LVDD	0.836	0.051	0.002	0.610	0.067	0.042
$LVMI_{BSA}$	0.015	0.055	0.002	-	-	-
LVH_{BSA}	0.444	0.015	0.118	−0.320	0.047	0.412
$LVMI_{height}$	0.031	0.062	0.001	0.030	0.051	0.253
LVH_{height}	0.530	0.021	0.062	−0.043	0.043	0.906

NT-proBNP, N-terminal pro-brain natriuretic peptide; hs-TnT, high-sensitivity cardiac troponin T; BMI, body mass index; kg, kilogram; m^2, square meter; WHR, waist to hip ratio; BPs, systolic blood pressure; mmHg, millimeters of mercury; BPd, diastolic blood pressure; HR, heart rate; bpm, beats per minute; A/G, android fat mass/gynoid fat mass; CPs, systolic central pressure; CPd, diastolic central pressure; PWV, pulse wave velocity; g, gram; HOMA-IR, homeostasis model assessment of insulin resistance; LVMI, left ventricular mass index; BSA, body surface area. Model 1: adjusted for $LVMI_{BSA}$.

Table S3. Multivariable predictors of left ventricular diastolic dysfunction.

**Variables**	**Model 4**		**Model 5**	
	**OR (95%CI)**	***p* values**	**OR (95%CI)**	***p* values**
Age, year	1.015 (0.977; 1.055)	0.438	1.013 (0.974; 1.053)	0.518
Gender, male	1.388 (0.657; 2.934)	0.390	0.821 (0.398; 1.692)	0.592
NT-proBNP, pg/mL	1.002 (1.000; 1.003)	0.011	1.002 (1.001; 1.003)	0.004
hs-TnT, pg/mL	1.035 (0.90; 1.082)	0.134	1.053 (1.000; 1.110)	0.051
BMI, kg/m^2	1.029 (0.968; 1.095)	0.358	1.025 (0.964; 1.089)	0.436
WHR *	1.013 (0.970; 1.057)	0.572	1.040 (0.996; 1.085)	0.076
BPs, mmHg	0.990 (0.973; 1.007)	0.260	0.993 (0.977; 1.010)	0.402

BPd, mmHg	0.990 (0.961; 1.020)	0.521	0.995 (0.966; 1.024)	0.725
A/G fat mass *	1.003 (0.989; 1.018)	0.648	1.009 (0.995; 1.023)	0.225
T- score	0.759 (0.578; 0.996)	0.047	0.766 (0.585; 1.003)	0.052
Z- score	0.747 (0.543; 1.029)	0.075	0.702 (0.508; 0.971)	0.033
Handgrip strength $_{max}$, kg	0.970 (0.941; 0.999)	0.045	0.982 (0.955; 1.011)	0.220
CPs, mmHg	0.986 (0.966; 1.006)	0.159	0.985 (0.966; 1.004)	0.129
CPd, mmHg	0.976 (0.945; 1.009)	0.154	0.981 (0.950; 1.013)	0.252
Augmentation Index	0.992 (0.956; 1.029)	0.665	0.975 (0.939; 1.013)	0.199
PWV, m/s	1.218 (1.002; 1.480)	0.047	1.270 (1.047; 1.540)	0.015
Diabetes mellitus	1.519 (0.795; 2.903)	0.206	1.467 (0.767; 2.805)	0.247
120 min glucose, mg/dL	1.008 (0.998; 1.018)	0.100	1.008 (0.999; 1.018)	0.096
HOMA-IR	1.073 (0.986; 1.167)	0.103	1.046 (0.962; 1.138)	0.290
LV ejection fraction, %	0.543 (0.444; 0.664)	<0.001	0.550 (0.450; 0.672)	<0.001

OR, odds ratio; CI, confidence interval; * per 0.1 units; NT-proBNP, N-terminal pro-brain natriuretic peptide; hs-TnT, high-sensitivity cardiac troponin T; BMI, body mass index; kg, kilogram; m^2, square meter; WHR, waist to hip ratio; BPs, systolic blood pressure; mmHg, millimeters of mercury; BPd, diastolic blood pressure; HR, heart rate; bpm, beats per minute; A/G, android fat mass/gynoid fat mass; CPs, systolic central pressure; CPd, diastolic central pressure; PWV, pulse wave velocity; g, gram; HOMA-IR, homeostasis model assessment of insulin resistance; LVMI, left ventricular mass index; BSA, body surface area; Model 4: adjusted for $LVMI_{BSA}$. Model 5: adjusted for LVH_{BSA}.
